# A mobile app for the treatment of female mixed and urgency incontinence: a cost-effectiveness analysis in Sweden

**DOI:** 10.1007/s00192-022-05137-1

**Published:** 2022-03-12

**Authors:** J. Ekersund, E. Samuelsson, L. Lindholm, M. Sjöström

**Affiliations:** Department of Public Health and Clinical Medicine, Umeå University, 905 81 Umeå, Sweden

**Keywords:** Urinary incontinence, e-health, Self-management, Health economics

## Abstract

**Introduction and hypothesis:**

A previous randomized controlled trial (RCT) demonstrated that the app Tät II, for self-management of mixed urinary incontinence (MUI) and urgency urinary incontinence (UUI), yielded significant, clinically relevant improvements in symptom severity and quality of life (QoL) compared with a control group. We aimed to assess the cost-effectiveness of Tät II.

**Methods:**

A cost–utility analysis with a 1-year societal perspective was carried out, comparing Tät II with an information app. Data were collected alongside an RCT: 122 community-dwelling women aged ≥18 years with MUI or UUI ≥2 times/week were randomized to 3 months of Tät II treatment focused on pelvic floor muscle training (PFMT) and bladder training (BT; *n* = 60), or to an information app (*n* = 62). Self-assessed data from validated questionnaires were collected at baseline and at 3-month and 1-year follow-ups. Costs for assessment, treatment delivery, incontinence aids, laundry, and time for PFMT and BT were included. We calculated quality-adjusted life-years (QALYs) using the International Consultation on Incontinence Modular Questionnaire Lower Urinary Tract Symptoms Quality of Life. The incremental cost-effectiveness ratio (ICER) between the groups was our primary outcome. Sensitivity analyses were performed.

**Results:**

The mean age was 58.3 (SD = 9.6) years. Annual overall costs were €738.42 in the treatment group and €605.82 in the control group; annual QALY gains were 0.0152 and 0.0037 respectively. The base case ICER was €11,770.52; ICERs in the sensitivity analyses ranged from €−9,303.78 to €22,307.67.

**Conclusions:**

The app Tät II is a cost-effective treatment method for women with MUI and UUI.

## Introduction

Urinary incontinence (UI) is defined as the complaint of involuntary loss of urine [1] and has a prevalence of about 25–45% among adult women [2]. There are three main types of UI: stress urinary incontinence (SUI), which is involuntary leakage of urine upon effort or physical exertion, sneezing, or coughing; urgency urinary incontinence (UUI), which is leakage associated with urgency; and mixed urinary incontinence (MUI) which is a combination of these symptoms [1]. SUI accounts for about half of all UI, followed by MUI and UUI respectively [2]. UI can have a negative impact on quality of life (QoL) and the impact increases with the severity of the leakage [3].

First-line treatment for all types of UI includes pelvic floor muscle training (PFMT). For MUI and UUI, bladder training is usually also recommended. As second-line treatment, pharmacological therapy can be considered [2, 4]. However, many affected women do not seek help. Embarrassment, shame, minimizing of problems, and a lack of knowledge about available efficient treatment can contribute to this [2].

Mobile technologies can be used for conservative self-management of UI and might increase satisfaction and adherence while reducing costs [5]. Women with MUI and UUI without alarm symptoms can be safely diagnosed and treated without face-to-face contact [6]. The eContinence project has developed the app Tät II for self-managed first-line treatment of female MUI and UUI, with a focus on PFMT and bladder training. The app has been evaluated in a randomized controlled trial (RCT) and shown to yield clinically relevant improvements regarding UI symptoms, urgency symptoms, and QoL [7].

Health economic evaluations are based on comparisons of the costs and effects of at least two treatment options and help to prioritize which treatments should be implemented. One commonly used method is the cost–utility analysis (CUA). A CUA can have either a health care perspective, which only includes costs borne by the health care system, or a societal perspective, where all costs are included regardless of who pays them [8]. In Sweden, the societal perspective is recommended [9]. The effect of treatment is defined by measuring QoL to calculate the change in quality-adjusted life-years (QALYs) [8]. The result is presented as an incremental cost-effectiveness ratio (ICER), and there are national [10] and international [11, 12] guidelines available to help evaluate the ICER.

Previous studies have evaluated the cost-effectiveness of internet-based and app-based treatment for SUI [13, 14], and for app-based treatment of SUI, MUI, and UUI compared with care as usual [15]. Here, we performed a cost–utility analysis of self-managed treatment of MUI and UUI, comparing the app Tät II with an app containing information but no treatment program.

## Materials and methods

This is a deterministic CUA with a 1-year societal perspective, performed according to the principles established by Drummond et al. [8].

### Study population and design

**Fig. 1:**
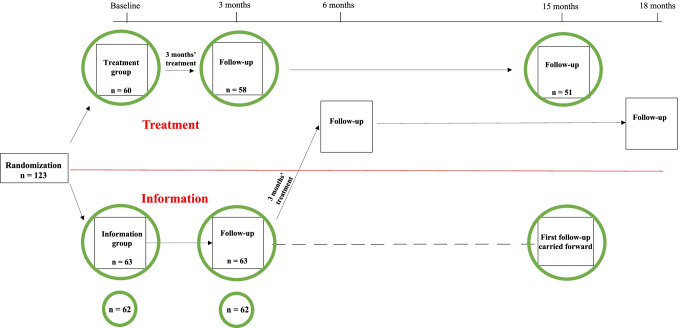
Flowcharts of the randomized controlled trial (*RCT*) and the cost–utility analysis (*CUA*). The boxes represent the RCT and the *green circles* represent the CUA. The timeline shows the different time points (baseline and follow-ups) of the RCT where data used in the CUA were collected. The area above the *red horizontal line* represents the treatment app, and the area below the line represents the information app. The *black arrow* crossing the *red line* represents the information group getting access to the treatment app after the 3-month follow-up. The *dashed line* shows that the information group’s 3-month follow-up data were also used for the 1-year follow-up

This CUA was based on data collected alongside an RCT evaluating the app Tät II, conducted in Sweden in 2017–2018, with full completion in early 2019. The RCT was registered at clinicaltrials.gov (ID:NCT03097549). [7]. The study flowchart of the RCT and the CUA is presented in Fig. 1.

The RCT’s study population consisted of 123 women with MUI or UUI, recruited via the research project’s website www.econtinence.se. Inclusion criteria were female sex, age ≥18 years, ≥2 or more leakages per week, symptom duration of ≥12 months, access to a smartphone, and ability to send and receive emails. Exclusion criteria included ongoing pregnancy, use of mirabegron or antimuscarinic drugs, use of another PFMT app, and UI surgery within the last 5 years. Other exclusion criteria concerned alarm symptoms indicating the need for physical examination and certain medical conditions: painful urges; previous pyelonephritis; ≥3 urinary tract infections in the last 12 months; dysuria; visible hematuria; uninvestigated bladder-emptying difficulties; metrorrhagia; cancer in the pelvic area, bladder, or bowels; decreased mobility or sensitivity in the legs or pelvic area; previous stroke; neurological disease; and diabetes. Assessment and diagnosis were based on self-completed web-based questionnaires (including validated symptom and QoL questionnaires, see below) sent by email to the participants, a 2-day bladder diary (frequency and measured volume of micturitions, and self-reported leakage episodes), and a telephone interview with a urotherapist to confirm the symptom diagnosis and the absence of exclusion criteria. There was no face-to-face contact with the participants at any time.

The participants were consecutively randomized to two groups. One group received the treatment app, Tät II (*n* = 60), which included an 11-step PFMT program prescribing training three times daily for 3 months, a seven-step bladder training program, psychoeducation, detailed information on each topic, general lifestyle advice, and individually tailored advice manually generated by a prespecified algorithm using information from each participant’s inclusion questionnaire and bladder diary. The development of the app and its included features have been described in detail elsewhere [7]. The other group (*n* = 63) received an app with brief information on each topic, but with no active treatment program. After 3 months of treatment, the treatment group was advised to maintain PFMT 2–3 times a week.

In this CUA, we included all participants from the RCT, except for one participant in the information group (total *n* = 122). The excluded participant had received an intravesical treatment for her UI symptoms prior to the 3-month follow-up.

### Data collection

Data collected at baseline included age, body mass index (BMI), education level, occupation, medication use, smoking, parity, current PFMT, use of incontinence aids, extra laundry due to UI, data on UI symptoms, and condition-specific QoL.

The first follow-up was performed 3 months (15 weeks) after randomization and included data on UI symptoms, QoL, use of incontinence aids, and laundry. The participants were also asked to estimate how much time they spent on PFMT and bladder training, and to what extent they did other things, such as walking, watching TV, etc., while performing the exercises. The second follow-up was performed 12 months later and included data on the same topics.

However, the information group was offered access to the treatment app after the 3-month follow-up. Thus, to obtain a control group for this CUA, we included 1-year follow-up data only for the treatment group, whereas for the information group we assumed that data from the 3-month follow-up would remain unchanged for the rest of the year.

### Symptom severity

Urinary incontinence symptoms were measured using the International Consultation on Incontinence Questionnaire - Urinary Incontinence Short Form (ICIQ-UI SF). The questionnaire consists of three scored items on frequency, amount of leakage, and the overall impact of UI on everyday life, summarized as an overall score of 0–21 points, with higher values indicating greater severity [16]. Based on the score, severity can be divided into the following categories: slight (1–5 points), moderate (6–12 points), severe (13–18 points), and very severe (19–21 points) [17].

### Costs

We included all relevant costs accrued over the course of the first year in the calculations.

Assessment costs included the estimated time spent by our study coordinator in emailing each participant web links to the questionnaires and the 2-day bladder diaries, and the estimated time for the telephone interview conducted by the urotherapist. Treatment delivery cost included the time spent by one of the researchers manually registering the tailored advice for each participant in the treatment group. The time costs were based on the employees’ average gross hourly wage including general payroll taxes in 2017.

We did not include the cost of app development. This is normally not included in health economic analyses, because it is a one-time cost comparable with, for example, the basic education cost of the health care personnel.

Participants’ time spent on PFMT and bladder training was calculated by combining their reported time spent on PFMT and bladder training with the estimates of how much of that time they spent on also doing other things, and we included the time spent exclusively on training. Pricing of the participants’ time was calculated based on average wages for women in Sweden with the same education level as our total study population [18]. The cost per unit was the gross hourly wage and included general payroll taxes.

The price of incontinence aids was based on the price of different absorbent pads for urine leakage protection as collected from the largest pharmacy chain in Sweden. From this, a mean price per unit, referring to a single pad, was calculated.

We estimated the cost of laundry based on the literature and national records [19]. It included the costs of electricity, electricity taxes, water consumption, laundry detergent, and washing machine wear and tear. The latter was based on the average price of a washing machine, assuming a lifespan of 10 years and an average of five washes per week. Participants’ time spent on managing a load of laundry was estimated to be 7.5 min.

At the 1-year follow-up, 4 participants had received pharmacological treatment for their UI and, owing to the low number, we did not include calculations of this cost.

All costs are presented in Euros at the 2017 year-end level. At that time, 1 EUR = SEK 9.85.

### Quality of life, utility weights, and quality-adjusted life-years

Condition-specific QoL was measured using the International Consultation on Incontinence Modular Questionnaire - Lower Urinary Tract Symptoms Quality of Life (ICIQ-LUTSqol) [20]. It consists of 19 items on different aspects of everyday life that might be affected by UI. Each item has four response choices: "never," "sometimes," "often," or "all the time," generating 1–4 points respectively. The values of the 19 items are summed to a total score of 19–76 points, with higher numbers indicating greater impact on QoL. We used an established index developed by Brazier et al. [21] to calculate QALYs from the questionnaire. The index incorporates 9 of the 19 items into a health state classification (a certain combination of items). Using the algorithms of the index, we then established syntax to translate the health state classifications into utility weights ranging from 0 (representing the worst) to 1 (the best imaginable health status).

### Primary outcome

Our primary outcome was the ICER. To calculate the ICER we used the following formula:

$$\mathrm{ICER}=\frac{{\mathrm{Cost}}_{\mathrm{Treatment}\ \mathrm{app}}-{\mathrm{Cost}}_{\mathrm{Information}\ \mathrm{app}}}{{\mathrm{QALY}}_{\mathrm{Treatment}\ \mathrm{app}}-{\mathrm{QALY}}_{\mathrm{Information}\ \mathrm{app}}}$$

### Statistics

For comparisons between groups, we used the Chi-squared test for categorical variables and Student’s *t* test for continuous variables. For analyses of improvement of QoL within each group, we used paired *t* tests. The total QALY change in each group was calculated using the “area-under-the curve,” whereas we considered costs to change linearly. *p* values <0.05 were considered statistically significant. We used SPSS Statistics (version 27.0; IBM Corp) for Mac and Excel for Mac (version 16.45; Microsoft Corp).

### Sensitivity analyses

We performed five univariate sensitivity analyses. In one, we adjusted the cost for one-third of our participants’ time by reducing the wages by 50% because they were retired. In the second, we halved the cost per unit of incontinence aids, considering that it might have been overestimated in our pricing, as women with UI presumably try to reduce the cost per unit by purchasing larger packages than those used in our first calculations. In the third we increased, and in the fourth we decreased, the time spent on PFMT and bladder training by 50%, thinking that there was a risk that the participants’ estimates of their time spent on training was either under- or overestimated in the base case. Finally, in the fifth, we removed the cost of participants’ time for training entirely. We also performed a multivariate sensitivity analysis, where we included the adjusted lower wage for the study population, the halved cost for incontinence aids, and the 50% lower value for the estimated time spent on training.

### Ethics

The study was approved by the Regional Ethical Review Board, Umeå University (number 2016/523-31). We obtained informed consent from all participants after they were thoroughly informed. No reimbursements were given.

## Results

### Study population

This study included 122 participants (treatment group *n* = 60, information group *n* = 62). Baseline characteristics of the two groups (Table 1), such as age, body mass index, UI symptoms, and QoL were similar. An exception was education level, as participants in the information group had a somewhat lower level of education.

**Table 1:** Baseline characteristics of the study population (*n* = 122)

	Treatment group (*n* = 60)	Information group (*n* = 62)	*p* value*
General information			
Age in years, mean (SD)	58.9 (9.2)	57.6 (9.9)	0.44
BMI in kg/m^2^, mean (SD)	26.5 (3.6)	26.0 (5.2)	0.58
University education ≥3 years, *n* (%)	44 (73.3)	34 (54.8)	0.03
Smoker, *n* (%)	1 (1.7)	2 (3.2)	**
Gynecology			
Nulliparity, *n* (%)	8 (13.3)	7 (11.3)	0.73
Postmenopausal ≥1 year, *n* (%)	42 (70.0)	46 (74.2)	0.61
Urinary incontinence			
ICIQ-LUTSqol^a^ score, mean (SD)	37.6 (8.3)	38.1 (8.2)	0.71
ICIQ-UI SF^b^ score, mean (SD)	11.7 (3.5)	11.5 (3.2)	0.66
ICIQ-UI SF score, severity category, *n* (%)			0.97
Slight, 1–5 points	2 (3.3)	1 (1.6)	
Moderate, 6–12 points	35 (58.4)	37 (59.7)	
Severe/very severe, 13–21 points	23 (38.3)	24 (38.7)	
Type of incontinence, *n* (%)			0.49
Mixed urinary incontinence	45 (75.0)	43 (69.4)	
Urgency urinary incontinence	15 (25.0)	19 (30.6)	
Daily users of incontinence aid, *n* (%)	23 (38.3)	26 (41.9)	0.69
Participants that run ≥1 extra laundry load per week due to UI, *n* (%)	31 (51.7)	32 (51.6)	0.995

*SD* standard deviation, *BMI* body mass index, *ICIQ-LUTSqol* International Consultation on Incontinence Questionnaire Lower Urinary Tract Symptoms Quality of Life

*ICIQ-UI SF* International Consultation on Incontinence Questionnaire - Urinary Incontinence Short Form

*p <0.05 was considered significant

**Not possible to analyze the statistical significance between groups owing to the small number of smokers in each group

^a^Higher values indicating greater impact on quality of life. Scoring scale: 19–76 points

^b^Higher values indicating greater severity of UI symptoms. Scoring scale: 0–21 points

After 3 months, 2 participants (3.3%) were lost from the treatment group, and none from the information group. Of the participants in the treatment group, 85% (51 out of 60) responded to the 1-year follow-up.

### Costs

The total assessment cost was €13.76 per participant in both groups. The treatment delivery cost (€2.42) was incurred only by the treatment group.

Participants in the treatment group had spent significantly more time on PFMT by the 3-month follow-up compared with participants in the information group (28.0 min vs 5.9 min per week, *p* <0.001). Thus, a higher cost for time spent on PFMT was seen in the treatment group compared with the information group (€330.83 vs €153.45), and for the treatment group this constituted the largest part of the total cost. The total annual cost was higher per participant in the treatment group (€741.62) than in the information group (€605.82; Table 2).

**Table 2:** Costs of the treatment group and information group, presented as annual costs per participant

	Price per unit	Units used		Costs	
	(€^e^)	Treatment group	Information group	Treatment group	Information group
Assessment^a^	13.76	1	1	13.76	13.76
Treatment delivery^b^	2.42	1	.	2.42	0
Participant costs					
Participant’s time^c^ for PFMT, mean (h)	30.14	10.98	5.09	330.83	153.45
Participant’s time for bladder training, mean (h)	30.14	6.14	3.95	184.89	119.13
Incontinence aids^d^, mean (*n*)	0.41	138.2	203.3	56.8	83.6
Extra laundry loads, mean (*n*)	0.68	34.4	53.0	23.4	36.1
Participant’s time for laundry, mean (h)	30.14	4.30	6.63	129.51	199.81
Total cost				741.62	605.82

*PFMT* pelvic floor muscle training

^a^Assessment cost includes the cost of time spent by the study coordinator and urotherapist on sending emails to and interviewing participants on the telephone respectively. These parts of the assessment were considered to be diagnosing urinary incontinence in a nontrial setting

^b^Includes the cost of time spent on manually registering tailored advice for the treatment group, generated by a pre-prepared algorithm

^c^Time for PFMT, bladder training, and laundry is presented as hours per year. The time unit for PFMT and bladder training presented in the table is adjusted by the time the women spent on doing other things while exercising. The pricing of participants’ time was based on the average hourly wage for women with the same education level as our study population

^d^Incontinence aids, referring to pads bought from a pharmacy. Presented as mean use per year

^e^All prices are presented in Euros (€), based on the 2017 year-end price

### Quality-adjusted life-years change

**Fig. 2:**
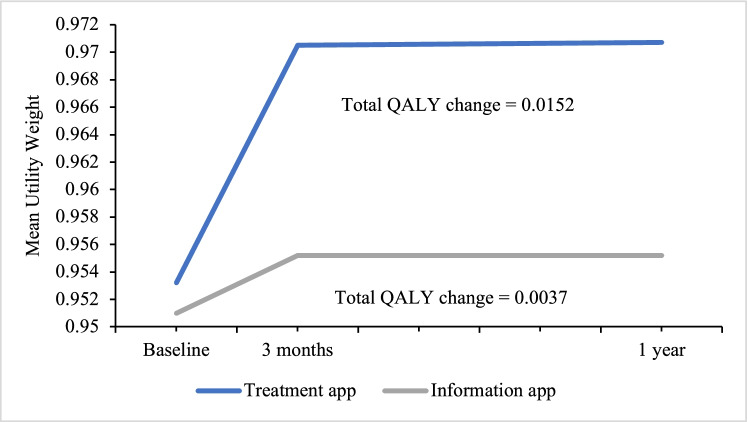
Total quality-adjusted life-years (*QALY*) change for the treatment app group and information app group. QALY change from baseline to 1-year follow-up. The timeline shows the three time points during the randomized controlled trial where data on participants’ quality of life were collected, using the International Consultation on Incontinence Questionnaire Lower Urinary Tract Symptoms Quality of Life (ICIQ-LUTSqol). The y-axis shows the mean utility weights, which were calculated based on results from the ICIQ-LUTSqol. The utility weights were then used to calculate the area under the curve for each graph, which represents the total QALY change over the year for each study group

Within both groups, there were significant improvements in QoL scores at the 3-month follow-up (mean ICIQ-LUTSqol reduction in the treatment group: 7.7, 95% CI 6.1–9.2) and mean ICIQ-LUTSqol reduction in the information group: 1.7, 95% CI 0.4–3.0). The improvement at the 3-month follow-up was maintained in the treatment group at the 1-year follow-up. The corresponding utility weights and QALY changes are presented in Fig. 2.

### Incremental cost-effectiveness ratio and sensitivity analysis

The ICERs for the base case and the sensitivity analyses are presented in Table 3. The ICER for the base case was €11,770.52. The treatment group had a higher total cost in all scenarios, but also larger QALY gains than the information group. The ICERs for the sensitivity analyses were within the range €−9,303.78 to €22,307.67, where the 50% increase and decrease in time spent on PFMT and bladder training resulted in the highest and lowest ICERs respectively.

**Table 3:** Incremental cost-effectiveness ratio (ICER) of the base case, and sensitivity analyses

	Total cost (€)^a^	QALY gain^b^	∆ Cost (€)	∆ QALY gain	ICER
Base case					
Treatment group	738.42	0.0152			
Information group	605.82	0.0037			
Treatment group vs information group			135.80	0.0115	11,770.52
Sensitivity analysis					
Univariate: wages reduced for the retired proportion of the participants					
Treatment group	641.13	0.0152			
Information group	532.25	0.0037			
Treatment group vs information group			108.88	0.0115	9,437.32
Univariate: cost of incontinence aids halved					
Treatment group	713.21	0.0152			
Information group	564.02	0.0037			
Treatment group vs information group			149.19	0.0115	12,930.70
Univariate: time for PFMT and bladder training reduced by 50%					
Treatment group	483.76	0.0152			
Information group	469.53	0.0037			
Treatment group vs information group			14.23	0.0115	1,233.37
Univariate: time for PFMT and bladder training increased by 50%					
Treatment group	999.48	0s.0152			
Information group	742.10	0.0037			
Treatment group vs information group			257.37	0.0115	22,307.67
Univariate: time for PFMT and bladder training removed entirely					
Treatment group	225.90	0.0152			
Information group	333.24	0.0037			
Treatment group vs information group			−107.34	0.0115	−9,303.78
Multivariate: wages reduced for the retired, cost of incontinence aids halved, time for PFMT and bladder training reduced by 50%					
Treatment group	395.02	0.0152			
Information group	375.39	0.0037			
Treatment group vs information group			19.63	0.0115	1,701.38

*PFMT* pelvic floor muscle training

^a^Costs presented in Euros (€) based on the 2017 year-end prices

^b^QALY gain over 1 year. Calculated as area under the curve

## Discussion

In this cost–utility analysis with a 1-year societal perspective, we demonstrate the cost-effectiveness of the app Tät II, which focuses on PFMT and bladder training for women with MUI and UUI, compared with an app containing information but no training programs. A higher overall cost was seen for the app Tät II, but also greater QALY gains than for the information app, resulting in a base case ICER of €11,770.52. The results were consistent in all tested scenarios.

### Strengths and limitations

One strength of this study is that the population can be considered clinically relevant, because all participants actively sought care for their UI, and 96.7% had moderate or severe leakage. Other strengths include the performance of the RCT by an experienced research group according to current guidelines, its low loss to follow-up, and its use of validated [16, 20] questionnaires. Moreover, most costs and QALYs were based on actual data collected from the participants, and we used an established method to calculate the QALYs [21]. Finally, our results were stable in all sensitivity analyses.

One limitation of this study is the comparison group. It would have been interesting to compare the treatment app with a Swedish care-as-usual alternative, but UI treatment varies considerably and there is no gold standard. Given that only a minority of women with UI seek help [2], we consider the comparison with the information app plausible, as it represents a good no-treatment alternative. Moreover, it is possible that participants with MUI experienced UUI symptoms to a variable degree at baseline and thus responded differently to therapy. However, the RCT design should have minimized the impact of this. Other limitations include the relatively small size of the study (*n* = 122), which may add uncertainty to our results, and the lack of 1-year data for the information group. Instead, based on our previous studies where effects have remained stable for 1 and 2 years [22, 23], we assumed that the information groups’ 3-month data would remain unchanged for the rest of the year. Moreover, the results may be difficult to generalize. The web-based recruitment might have led to a selection bias in the study population. Internet use is widespread in Sweden; for example, in 2018, 90% of Swedish women aged 16–74 years used the internet daily [24]. Nevertheless, our population had a higher education level (63.9% had a university education ≥3 years) than the average Swedish female population in 2018; 30.5% of women aged 25–74 years had the same education level as those in our study [25]. However, it has previously been shown that education level does not seem to affect the ability to learn to perform PFMT [26].

### Comparison with prior work

The total annual cost was higher in the treatment group than in the information group (€738.42 vs €605.82 respectively). This can be compared with the overall annual cost per participant in our previous CUAs; one compared an internet-based program to a postal treatment program for SUI (overall costs €596.50 and €596.20 respectively) [13], and one assessed the cost-effectiveness of self-management of SUI using the app Tät (overall cost €547.00) [14]. The largest cost in these studies was the participants’ time spent on PFMT, but as both focused on the treatment of SUI, neither included any time for bladder training. For further comparison, Loohuis et al. recently reported overall costs of app-based treatment for SUI, MUI, and UUI, using a 1-year societal perspective, of €1,520, and a cost of care-as-usual of €1,680 [15]. Moreover, from the OPAL study including women with SUI and MUI receiving basic PFMT, Hagen et al. presented an overall cost in the basic PFMT group of £1,535.26 (≈ €1,790) when applying a societal perspective for 24 months [27]. Pharmacological treatment, which is the recommended second-line treatment, is considerably more expensive. For example, Visco et al. presented a comparison of anticholinergics and intradetrusor Botox injections in the treatment of UUI, with annual per patient costs of $2,978 (€2,453) and $2,744 (€2,260) respectively [28].

The treatment group in our study had a total QALY gain of 0.0152, which can be translated into 5.5 additional days in the best imaginable health status for 1 year. The QALY gain observed in the treatment group was higher than that provided by an internet-based program focusing on PFMT for treatment of SUI (QALY gain 0.0104) [13], and for the app Tät, which is intended for self-management of SUI (QALY gain of 0.0101) [14]. It was also higher than the incremental QALY gains (0.01) reported by Albers-Heitner et al. from a primary care setting where women with SUI performed PFMT under the guidance of a specialist nurse or in a GP care-as-usual alternative [29]. The larger QALY gains in our study were expected, since MUI and UUI often have a greater negative impact on QoL than SUI [3, 30], and thus there is a larger potential for improvement. The QALY gain for the treatment group was lower than from pharmacological treatment, where QALY gains of 0.046 and 0.039 for anticholinergic and Botox treatment have been presented [28]. However, although the QALY gains in the treatment group might seem small, both MUI and UUI are common conditions and many women may be helped by the treatment; thus, on a population level, they imply a considerable effect.

The ICER for the base case was €11,770.52, whereas the ICERs for the sensitivity analyses were in the range €−9,303.78 to 22,307.67. Whether this represents cost-effectiveness or not depends on the willingness to pay for extra QALYS, and this might vary in different countries. In Sweden, an incremental cost per QALY gained of less than €10,000 is considered low, and a cost of €49,500–€99,000 is considered high [10]. In the UK, the NICE guidelines prescribe an additional cost for a new intervention in the range €22,500–34,000 (£20,000–30,000) per QALY gained to be considered cost-effective [11], whereas in the US, at least €41,000 ($50,000) per QALY gained is suggested as a threshold value for cost-effectiveness [12].

### Clinical implication and future research

There is a need for cost-effective and easily accessible treatment methods for MUI and UUI, and this study provides additional knowledge for guidance when prioritizing what methods should be implemented in clinical practice. Although app treatment might not be the most suitable method for all women, Tät II could provide a new first-line treatment option for many affected women. However, this CUA was performed in a study setting, and when the app is used in other populations, costs and effects are likely to change. Thus, further evaluation of its cost-effectiveness when made publicly available would be of interest. Most likely, the overall costs will be lower in another population, which is not as highly educated as ours, resulting in a reduced cost for the participants’ time. Furthermore, the cost of PFMT would probably be reduced over time, as the women increasingly learn how to train while doing other things. On the other hand, costs of maintenance and technical support of the app will need to be added, but those costs will be split among many users.

## Conclusions

The app Tät II is a cost-effective treatment method for women with MUI and UUI. It can be considered a new, first-line treatment option, and may be a way to reach more women wishing treatment.
